# Relationship between fetal-type posterior cerebral artery and basilar artery atherosclerosis

**DOI:** 10.3389/fneur.2025.1533281

**Published:** 2025-03-19

**Authors:** Lijuan Zheng, Yaozhang Chen, Xin Lin, Shasha Deng, Bin Sun, Jinmei Zheng, Fang Zeng, Yunjing Xue

**Affiliations:** ^1^Department of Radiology, Fujian Medical University Union Hospital, Fuzhou, China; ^2^School of Medical Imaging, Fujian Medical University, Fuzhou, China; ^3^Minimally Invasive Surgery Training Center of Fujian Medical University Union Hospital, Fuzhou, China; ^4^Department of Neonatology, Fujian Maternity and Child Health Hospital, College of Clinical Medicine for Obstetrics & Gynecology and Pediatrics, Affiliated Hospital of Fujian Medical University, Fuzhou, China; ^5^The School of Medical Imaging, Changsha Medical University, Changsha, China

**Keywords:** posterior cerebral artery, basilar artery plaque, morphology factor, ischemic stroke, relationship

## Abstract

**Objective:**

To investigate the relationship between the morphology of posterior cerebral artery (PCA) and the basilar artery (BA) atherosclerosis disease based on the High-Resolution Magnetic Resonance Vessel Wall Imaging (HR-MRI).

**Methods:**

A total of 321 patients presented with cerebrovascular symptoms (posterior circulation ischemic stroke or transient ischemic attack <2 weeks) at the Department of Neurology were collected from July 2017 to June 2020. We systematically collected clinical information, encompassing demographics, medical histories (smoking, alcohol consumption, diabetes, hypertension, hyperlipidemia, and coronary heart disease), and relevant mediation histories. BA curvature, mean lumen area, mean normalized wall index and the morphology of PCA were accessed with HR-MRI and magnetic resonance angiography. The binary logistic regression analysis was used to identify the risk factors of BA plaque formation. Spearman’s bivariate method and correlation coefficients were calculated to analyze the correlations between the morphology of PCA and BA plaque burden. The relationship between different PCA morphologies and posterior circulation infarction was analyzed by Chi square test.

**Results:**

Hypertension, diabetes and fetal-type posterior cerebral artery (FTP) were independent risk factors for BA plaque formation in Walking and Lambda geometry subtypes (*p* < 0.05). For the parameters of BA plaque burden, the PCA morphological type was positively correlated with Mean NWI (*r* = 0.252, *p* = 0.03), and that was negatively correlated with mean lumen area (*r* = −0.35, *p* = 0.002) and mean vessel area (*r* = −0.275, *p* = 0.018) in Lambda subtype. The incidence rate of posterior circulation infarction was statistically significant among different PCA morphologies (*p =* 0.018).

**Conclusion:**

FTP was a risk factor of BA plaque formation and it was correlated with BA burden, and which could be used to explain the posterior circulation infarction in patients with FTP.

## Introduction

Basilar Artery (BA) atherosclerosis (1, 2), a primary cause of posterior circulation ischemic stroke, is defined as an inflammatory condition affecting the arterial wall. It is largely believed to result from a confluence of factors, including smoking, hypertension, hyperlipidemia, diabetes, vascular morphology, and hemodynamics (3–5). The vascular morphology and hemodynamics of BA system were considered as the most important and unique factors in BA atherosclerosis (6, 7). However, the variations in the PCA have been less frequently discussed in studies on posterior circulation.

The Fetal-type posterior cerebral artery (FTP), is a vascular structure with an origin (P1 segment) that deviates from the typical physiological anatomy, representing one of the most common variations in the posterior circulation (8). The PCA is categorized into three distinct types based on the diameter of the P1 segment and the size of the ipsilateral Posterior Communicating Artery. These types include the Normal PCA, Complete Fetal-type Posterior Cerebral Artery (CFTP), and Partial Fetal-type Posterior Cerebral Artery (PFTP). The presence of the FTP significantly influences the local hemodynamic forces within both the anterior and posterior circulations (9), leading to alterations in hemodynamic parameters such as blood flow distribution, velocity, and vascular wall pressure (10). These hemodynamic alterations are crucial in the development of intracranial atherosclerotic diseases. Previous research have indicated that individuals with FTP have an increased risk of anterior circulation ischemic stroke, attributed to hemodynamic changes within the internal carotid artery system (11, 12). Additionally, FTP patients exhibit reduced basilar artery (BA) flow volume and a smaller BA diameter, which complicates BA hemodynamics and increases flow resistance (6, 13).

Drawing from the existing body of research, we hypothesize that the morphology of the PCA is associated with BA atherosclerosis. Consequently, our objective is to scrutinize the correlation between PCA morphology and the incidence of posterior circulation ischemic stroke.

## Materials and methods

### Study population

This study was conducted in our center (Ethics approval number: 2019KJTZD009), and it is the largest comprehensive stroke treatment hospital in Fujian Province, China. Patients presented with cerebrovascular symptoms (posterior circulation ischemic stroke or transient ischemic attack <2 weeks) at the Department of Neurology were collected for retrospective analysis from July 2017 to June 2020. We systematically collected comprehensive clinical information for these patients, encompassing demographics (name, gender, age, BMI), medical histories (smoking, alcohol consumption, diabetes, hypertension, hyperlipidemia, and coronary heart disease), and the relevant medication history (warfarin, enteric-coated aspirin, metoprolol, clopidogrel hydrogen shlfate tablet, amlodipine besylate tablet). The inclusion criteria were as follows: (I) Patients with posterior circulation ischemia. (II) Completion of routine Magnetic Resonance Imaging (MRI) including Diffusion Weighted Imaging (DWI), Three-Dimensional time-of-flight magnetic resonance angiography (3D TOF-MRA), and 3D T1 CUBE (HR-MRI) examinations within two weeks. (III) Patients informed consent. The exclusion criteria were as follows: (I) Patients with anterior circulation infarction. (II) Non-atherosclerotic vascular disease, such as BA dissection, Moyamoya disease, and vasculitis. (III) Cardiogenic stroke (atrial fibrillation, post-valve replacement, endocarditis). (IV) BA or VA occlusion, the location of BA was from the top of the BA to the junction of both vertebral arteries (14), Patients with basilar and vertebral artery occlusion did not exist the junction of both vertebral arteries, so we excluded them. (V) the quality of MR images ≤ grade 2.

### Imaging protocol

All patients were completed the MRI examinations within 1-2d after hospitalization by 3.0 T MR system (Discovery 750w, GE Healthcare, Milwaukee, WI, USA) with a 32-channel head coil. MRI sequences included DWI, 3D TOF-MRA and 3D T1 CUBE images. DWI: FOV = 230 mm × 230 mm, thickness = 5.0 mm, matrix = 128 × 128, B value = 1,000, NEX = 3; 3D TOF-MRA: TR/TE = 20/3.4 ms, FOV = 180 mm × 180 mm, flip angle = 20°, thickness = 1.0 mm, matrix = 512 × 192, acquisition time = 4 min 23 s; 3D T1-CUBE: TR/TE = 600/14 ms, FOV = 200 mm × 160 mm, thickness = 0.8 mm, matrix = 320 × 288, bandwidth = 50 Hz, echo train length = 28, acquisition time = 6 min 31 s. The acquired coronal 3D T1-CUBE images were imported into a post-processing workstation (Advantage Workstation, AW4.5; GE Medical Systems) for axial reconstruction with a layer thickness of 0.8 mm and a layer spacing of 0 mm.

### Morphological analysis

**Figure 1:**
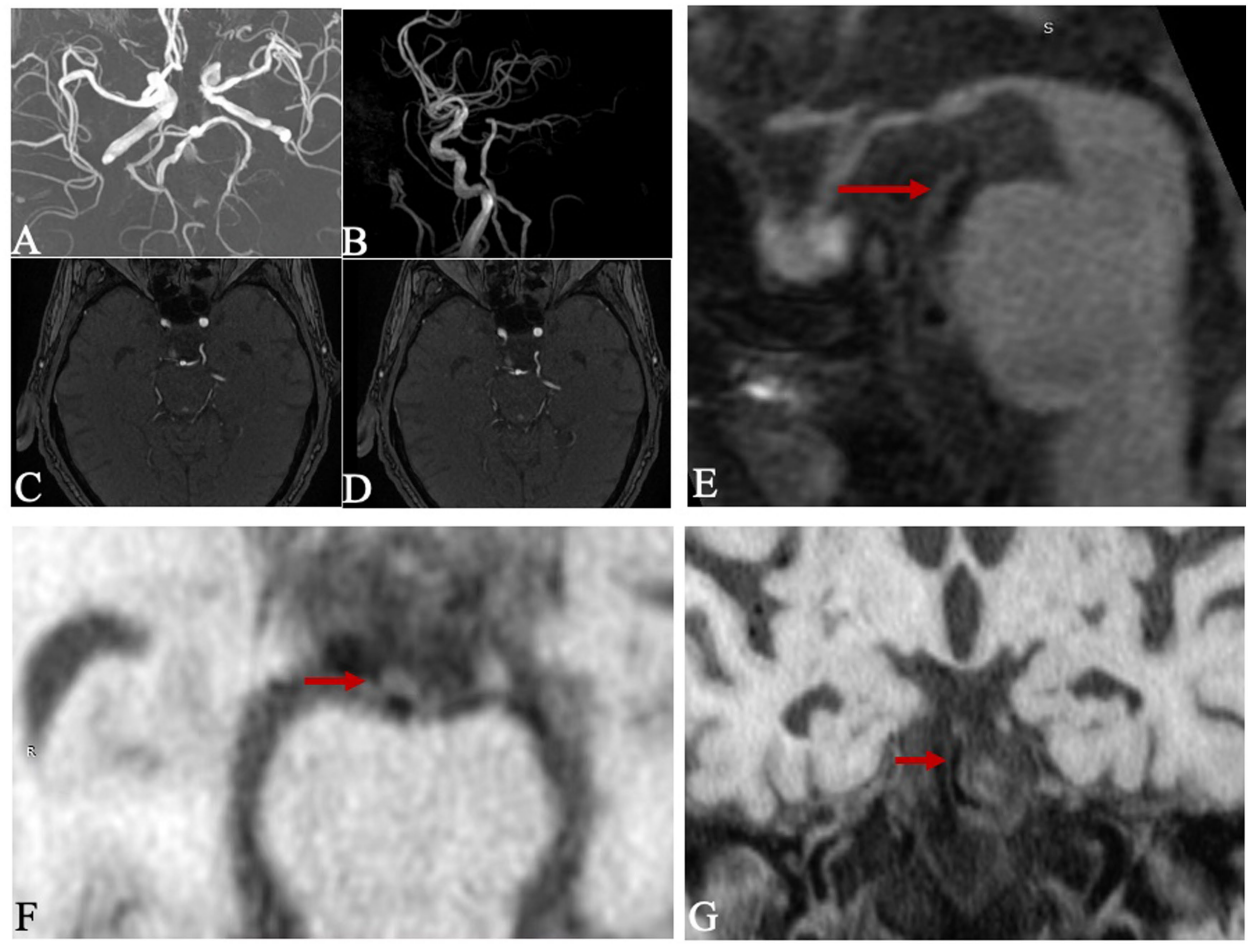
Anatomical morphology of the vertebral artery and PCA on 3D TOF-MRA: the morphology of vertebral artery was categorized as Walking **(A)**, Tuning Fork **(B)** and Lambda **(C)** Geometries according to the diameter difference and bend direction in bilateral vertebral artery. The morphology of PCA was categorized as normal PCA **(D)**, CFTP **(E)** and PFTP **(F)** according to the development of PI segment.

3D TOF-MRA was used to determine the vertebral artery anatomies and PCA variation. The anatomical configuration of the vertebrobasilar artery included three geometries (15). Walking geometry refers to the bilateral vertebral arteries have similar diameters (<0.3 mm) and coverage in same bending direction to form BA; Tuning fork geometry refers to the bilateral vertebral arteries have similar diameters (<0.3 mm) and coverage in opposite bending direction to form BA; Lambda geometry refers to the bilateral vertebral arteries have different diameters (≥0.3 mm) to form BA. The PCA morphology included three geometries. Normal PCA refers to the vessel outer diameter of P1 segment is larger than the ipsilateral posterior communicating artery (PcoA), with a PcoA/P1 < 1, and its blood mainly originates from BA system; CFTP refers to the P1segment is disappeared, and its blood is all from the ipsilateral internal carotid artery (ICA); PFTP means that the P1 segment is visible on MRI, but the outer diameter of P1 segment is significantly smaller than the ipsilateral PcoA, with a 1 < PcoA/P1 < 1.5, and its blood mostly comes from the ipsilateral ICA (9, 16). It was shown in Figure 1.

### BA plaque burden analysis

**Figure 2:**
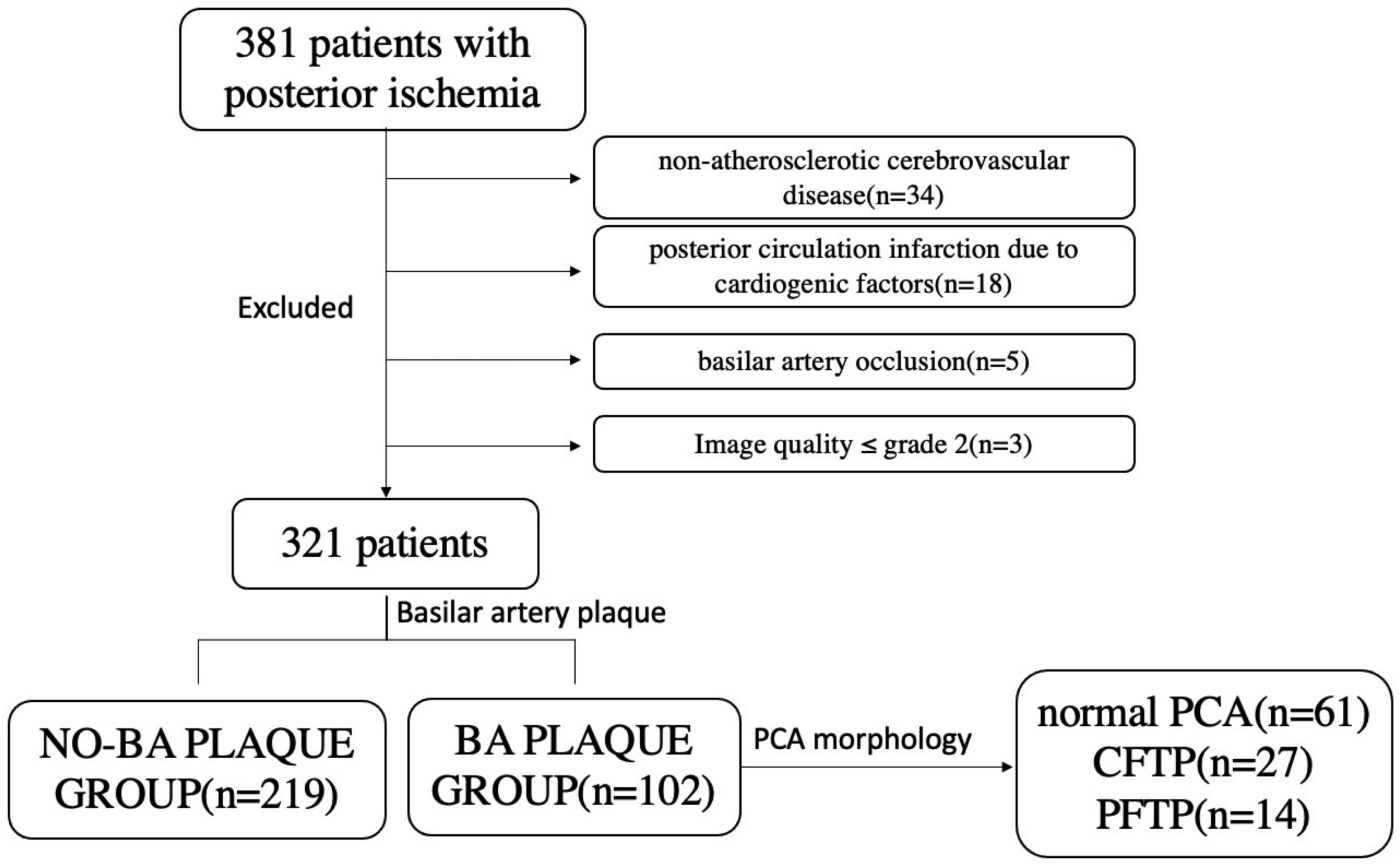
Example figures of CFTP on 3D TOF-MRA, and the plaque on 3D CUBE T1W1. Images **(A–G)** were from a 78-year-old male patient. **(A–D)** were reconstructed images and presented as a fetal posterior cerebral artery. There was an atherosclerotic plaque (red arrow) on the distal of basilar artery on sagittal image **(E)**, corresponding reconstructed axial image **(F)** and coronal image **(G)**.

The 3D T1-CUBE images were analyzed by two physicians with more than three years’ experience in diagnostic imaging of the arterial wall by using Vessel explorer 2.0 (TSimaging Healthcare, Beijing, China) software. The main observations were provided as follows: ① Basilar atherosclerotic plaque (17) refers to limited, eccentric wall thickening, with the thinnest part of the wall being less than 50% of the thickest part. As shown in the Figure 2, the plaque at the distal of BA is clearly on 3D T1-CUBE images. ② BA curvature (14): (true length of BA/straight length of BA - 1) *100; ③ Plaque burden (18): Mean normalized wall index (Mean NWI), which is the ratio of wall area to total vessel area. Mean lumen area and Mean NWI are accurate and repeatable parameters. These were commonly used to indicate the burden of plaque.

### Statistical analysis

The statistical analysis was performed by SPSS 26.0 (IBM Corp, Armonk, NY, USA). The measurement data were expressed as mea*n* ± SD and analyzed by one-way ANOVA or t-test methods. Continuous data were analyzed using *t*-test (ANOVA test) or the Mann–Whitney U test method. Categorical variables were tested by the chi-square test or Fisher’s exact test. After adjusting the confounders, a binary logistic regression model was developed to investigate the correlation between the morphology of PCA and BA plaque using forest plots and ratio ratios. Correlations between the morphology of PCA and the burden of BA plaque were analyzed by Spearman’s bivariate method and correlation coefficients (−1 < *r* < 0 indicates negative correlation; *r* = 0 indicates zero correlation; 0 < *r* < 1 indicates positive correlation). *p < 0.05* was statistically significant.

## Results

### Study population

**Figure 3:**
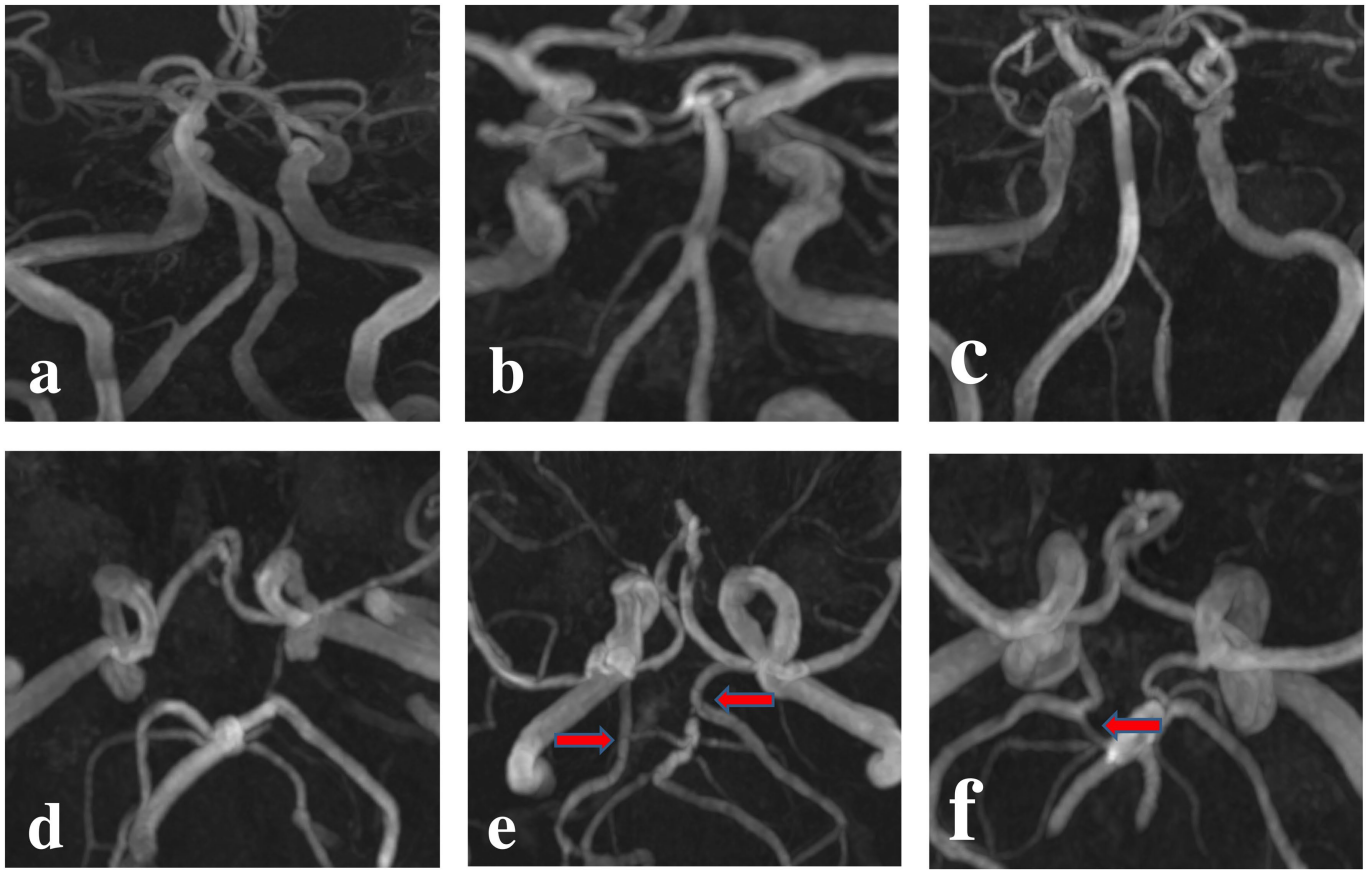
Flow diagram for patient population of posterior ischemia.

A total of 321 patients were eventually enrolled. Patients were divided into the BA plaque group (*n* = 102) and no-BA plaque group (*n* = 169). Each group was further divided into three subgroups based on geometric criteria: Walking Geometry, Lambda Geometry, and Tuning Fork Geometry. The BA plaque group was further divided into the normal PCA, CFTP, and PFTP groups according to the morphology of the PCA, as shown in Figure 3. A comparative analysis of patients with and without BA plaque across the two groups, as presented in Table 1, revealed no significant differences in terms of age, sex, and body-mass index (*p > 0.05*).

**Table 1:** Univariate analysis of basilar artery plaque.

Subgroup	BA plaque	No-BA plaque	Estimate	CI low	CI high	*p* Value
L geometry	74	169				
Male	45	103	0.439	0.568	1.740	<0.001
Age	71.1 ± 1.2	66.6 ± 0.8	1.041	1.013	1.069	0.004
Body-mass index	23.9 ± 0.4	23.5 ± 0.3	1.040	0.954	1.134	0.373
Smoking	29	63	1.084	0.619	1.901	0.777
Hypertension	64	106	3.804	1.822	7.939	<0.001
Diabetes	31	55	1.494	0.851	2.623	0.162
Hyperlipidemia	21	52	0.892	0.488	1.627	0.708
Coronary heart disease	4	21	0.403	0.133	1.218	0.107
PCA aberration rate	33	34	3.196	1.766	5.782	<0.001
W geometry	18	20				
Male	12	12	1.333	0.354	5.026	0.671
Age	70.5 ± 2.1	75.4 ± 1.6	0.928	0.853	1.009	0.081
Body-mass index	24.1 ± 0.6	25.1 ± 0.9	0.910	0.733	1.131	0.397
Smoking	6	8	0.750	0.199	2.827	0.671
Hypertension	15	12	2.692	0.575	12.596	0.208
Diabetes	13	4	10.400	2.310	46.831	0.002
Hyperlipidemia	6	2	4.500	0.775	26.133	0.094
Coronary heart disease	3	4	0.800	0.153	4.184	0.792
PCA aberration rate	7	1	12.091	1.309	111.657	0.028
T geometry	10	30				
Male/Female	4	17	0.510	0.119	2.188	0.365
Age	69.9 ± 4.9	66.2 ± 1.7	1.021	0.960	1.087	0.506
Body-mass index	22.9 ± 1.1	23.3 ± 0.6	0.959	0.755	1.218	0.753
Smoking	1	9	0.259	0.028	2.360	0.231
Hypertension	9	19	5.211	0.580	46.808	0.141
Diabetes	5	13	1.308	0.312	5.490	0.714
Hyperlipidemia	4	6	2.667	0.566	12.557	0.215
Coronary heart disease	1	4	0.722	0.071	7.340	0.783
PCA aberration rate	1	7	0.365	0.039	3.404	0.376

### Morphological analysis

**Figure 4:**
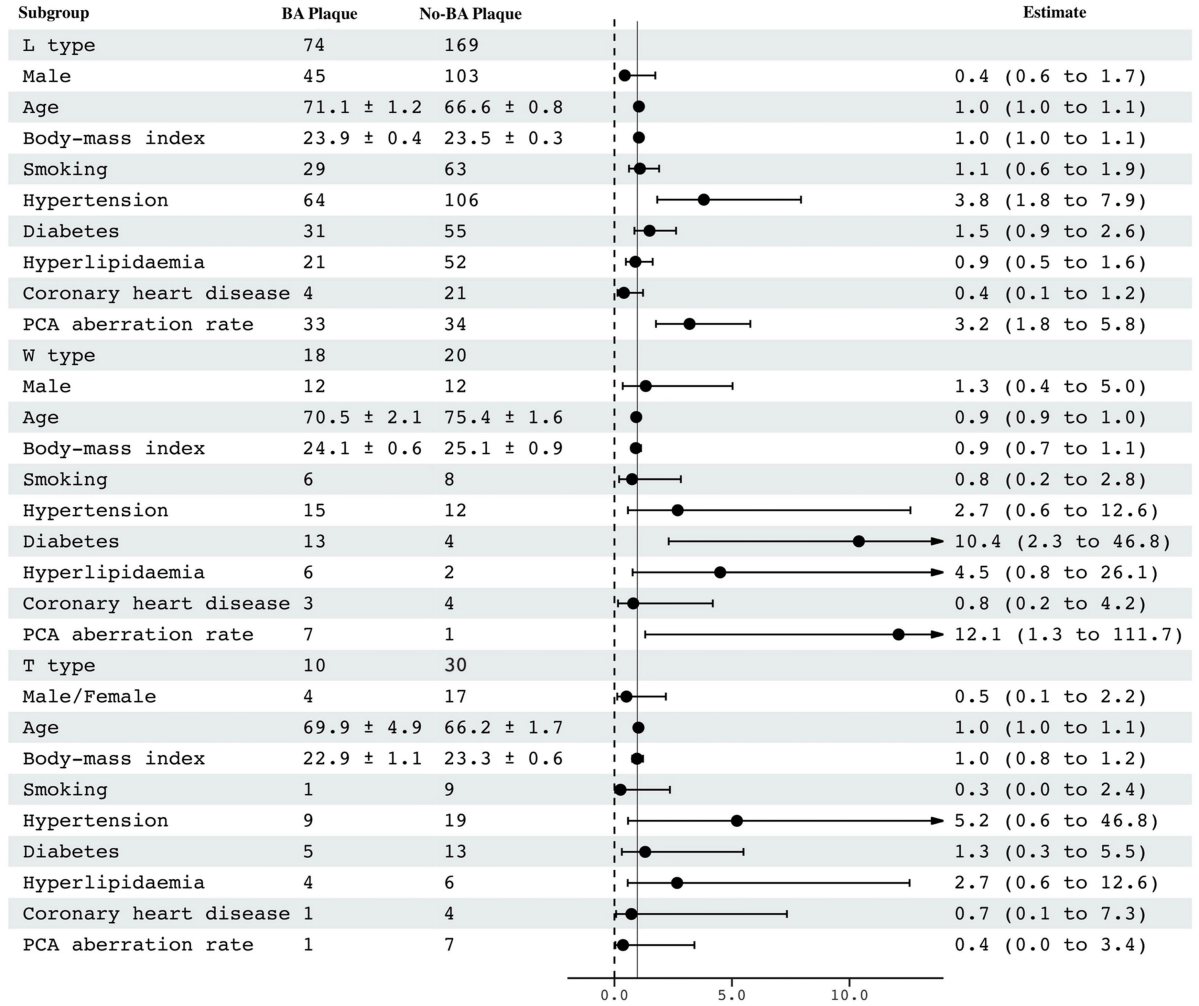
Analysis of risk factors for plaque formation in basilar arteries.

The clinical parameters and PCA morphology for the two groups were listed in Table 1 based on three vertebrobasilar geometries. It was shown that sex, age, hypertension, and FTP were risk factors for BA plaque formation in patients with Lambda geometry (*p < 0.05*). In patients with Walking geometry, diabetes and FTP were risk factors for BA plaque formation (*p < 0.05*). After adjusted (*VIF < 3*), we found that hypertension, diabetes and FTP were independent risk factors (*p < 0.05*). It showed that the incidence rate of BA plaque formation in the hypertensive patients was 3.8 times higher than that in the normotensive patients (95% CI: 1.82–7.94). The incidence rate of BA plaque formation was increased up to 10.4 times in the patients with diabetes compared to that in the patients with normal blood glucose (95% CI: 2.31–46.83). The FTP, as indicated PCA aberration rate, had a significant difference between patients in the group of BA plaque and no-BA plaque (*p < 0.05*) both in Lambda and Walking geometries. However, we have not found any risk factors for the BA plaque formation among the patients with Tuning Fork geometry (*p > 0.05*), as shown in Figure 4.

After being analyzed the relevant medication history of patients with varying vertebrobasilar artery structural morphologies between BA plaque and No-BA plaque groups, we found there were not any differences between them (all *p > 0.05*), as shown in Table 2.

**Table 2:** The relevant medication history of patients with varying vertebrobasilar artery structural morphologies between BA plaque and No-BA plaque groups.

Subgroup	BA plaque	No-BA plaque	*P* Value
L geometry			
Warfarin	0/63	6/149	0.185 ^b^
Enteric-coated aspirin	24/39	44/111	0.161
Metoprolol	3/60	10/145	0.871^a^
Clopidogrel hydrogen sulfate tablet	36/27	103/52	0.195
Amlodipine besylate tablet	12/50	20/133	0.241
W geometry			
Warfarin	0/9	0/27	—
Enteric-coated aspirin	4/5	8/19	0.683 ^a^
Metoprolol	0/9	2/25	0.557 ^b^
Clopidogrel hydrogen sulfate tablet	7/2	15/12	0.430 ^a^
Amlodipine besylate tablet	1/8	5/22	1.000 ^a^
T geometry			
Warfarin	0/15	0/19	—
Enteric-coated aspirin	8/7	5/14	0.107
Metoprolol	1/14	1/18	1.000 ^a^
Clopidogrel hydrogen sulfate tablet	13/2	16/3	1.000 ^a^
Amlodipine besylate tablet	4/11	1/18	0.207 ^a^

^aCorrected Chi square test; bFisher’s test.^

### BA plaque burden analysis

The analysis of BA plaque burden was carried out for 102 patients. Table 3 showed that the PCA morphological type was negatively correlated with the BA plaque burden parameters in patients with Lambda geometry, including mean lumen area (*r* = −0.35), mean vessel area (*r* = −0.275), and mean lumen diameter (*r* = −0.292), and that was positively correlated with Mean NWI (r = 0.252) and BA curvature (*r* = 0.429), *p < 0.05*. In the patients with Walking geometry, the PCA morphology type was negatively correlated with BA plaque burden parameters, including mean BA lumen area (*r* = −0.532), mean vessel area (*r* = −0.606), maximum wall thickness (*r* = −0.506), and mean vessel diameter (*r* = −0.738), *p < 0.05*. Not any relationships were found between the PCA morphological type and BA plaque burden in patients with Tuning fork geometry (*p > 0.05*), as shown in Table 3.

**Table 3:** The relationship between posterior cerebral artery geometry and the burden of plaque.

Parameters	Mean LA	Mean WA	Mean VA	Mean WT	Mean NWI	Mean max WT	Average profile area of BA	Curvature of BA
L geometry								
Normal PCA	7.6 ± 0.4	15.4 ± 0.5	23.0 ± 0.7	1.1 ± 0.03	67.1 ± 1.1	2.5 ± 0.06	3.8 ± 0.1	11.4 ± 0.8
PFTP	6.0 ± 0.9	14.6 ± 1.5	20.5 ± 2.0	1.1 ± 0.08	71.0 ± 2.7	2.4 ± 0.10	3.4 ± 0.2	19.0 ± 2.0
CFTP	6.5 ± 0.8	15.1 ± 0.9	21.6 ± 1.7	1.2 ± 0.04	71.5 ± 1.6	2.4 ± 0.07	3.6 ± 0.1	17.9 ± 2.5
*r*	−0.35	−0.107	−0.275	0.107	0.252	−0.058	−0.292	0.429
*P* value	0.002	0.366	0.018	0.362	0.030	0.621	0.012	<0.001
W geometry								
Normal PCA	8.6 ± 0.6	17.0 ± 1.4	25.6 ± 1.6	1.1 ± 0.08	65.8 ± 2.3	2.5 ± 0.12	4.0 ± 0.1	14.2 ± 3.6
PFTP	5.7 ± 1.0	11.8 ± 1.5	17.5 ± 2.3	1.0 ± 0.07	68.2 ± 3.3	2.0 ± 0.10	3.2 ± 0.1	13.4 ± 3.6
CFTP	5.6 ± 1.8	15.1 ± 0.9	20.7 ± 2.7	1.2 ± 0.04	74.6 ± 3.9	2.6 ± 0.02	2.8 ± 0.4	14.5 ± 5.0
*r*	−0.523	−0.448	−0.606	−0.145	0.274	−0.506	−0.738	0.108
*P* value	0.026	0.062	0.008	0.565	0.272	0.032	<0.001	0.670
T geometry								
Normal PCA	6.6 ± 0.6	13.2 ± 0.9	19.8 ± 1.2	1.0 ± 0.1	66.8 ± 2.2	2.3 ± 0.1	3.8 ± 0.2	9.1 ± 1.6
PFTP	Na	Na	Na	Na	Na	Na	Na	Na
CFTP	Na	Na	Na	Na	Na	Na	Na	Na
*r*	−0.406	0.290	−0.290	0.407	0.406	0.406	−0.290	0.290
*P* value	0.244	0.416	0.416	0.243	0.244	0.244	0.416	0.416

PCA, posterior cerebral artery; PFTP, partial fetal posterior cerebral artery; CFTP, complete posterior cerebral artery; mean LA, mean lumen area; mean WA, mean wall area; mean VA, mean vessel area; mean WT, mean wall thickness; mean NWI, mean normalized wall index; mean max WT, mean maximum wall thickness; max NWI, maximum normalized wall index; min LA, minimum lumen area; mean area of basilar artery; mean true BAL, the true value of mean basilar artery length; mean BAL, the value of mean basilar artery straight length; mean tor, mean tortuosity of basilar artery; L geometry, Lambda geometry; W geometry, Walking geometry; T geometry, Tuning fork geometry.

### Infarction among PCA morphologies

There were 21.8% patients (70 of 321) developed acute posterior circulation infarction while 78.2% patients (251 of 321) developed transient ischemic attack (TIA) in the posterior circulation. There were significant differences in cerebral infarction among the three groups, including NO-PCA (18.1%), PFTP (34.5%) and CFTP (31.5%) (*p = 0.022*), as shown in Table 4.

**Table 4:** The relationship between fetal posterior cerebral artery and Posterior circulation infarction.

	TIA	Infarction	χ^2^	*P*
Normal PCA	195 (77.6%)	43 (61.4%)	7.649	0.018
PFTP	19 (7.6%)	10 (14.3%)		
CFTP	37 (14.7%)	17 (24.3%)		

PCA, posterior cerebral artery; PFTP, partial fetal posterior cerebral artery; CFTP, complete posterior cerebral artery; TIA, transient ischemic attack.

## Discussion

In patients with FTP, the blood supplying to the posterior cerebral artery predominantly originates from the internal carotid artery system. This anatomical variation can lead to chronic overload on the vessel walls, promoting thrombosis and increasing the risk of anterior circulation ischemic stroke (19). Previous studies (20) identified the presence of the FTP structure as a risk factor for anterior circulation ischemic stroke (OR = 3.027), but did not specifically investigate its role in posterior circulation ischemic stroke. Our team is the first to propose a relationship between FTP and posterior circulation cerebrovascular diseases, addressing this gap in the literature. The FTP structure in our study, as the variation of PCA, exerted a significant impact on the blood flow distribution within the BA system. This effect was particularly pronounced in patients with the FTP structure who exhibited an abnormal BA blood flow pathway due to the P1 segment on one side of the PCA. Consequently, the contralateral PCA had to accommodate the entire blood flow from the BA system, resulting in an increased average profile area of the BA compared to normal vessels (*p = 0.012*). This alteration in hemodynamics led to elevated intravascular pressure, which in turn increased the risk of atherosclerosis in posterior circulation ischemic cerebrovascular diseases. The detailed relationship between the FTP and posterior circulation will be further elucidated in the subsequent discussion.

It has been demonstrated that the morphology of the VA can be classified into three distinct categories: Walking, Lambda, and Tuning Fork geometries. Patients exhibiting Walking and Lambda geometries are at an elevated risk for the development of BA plaques (21). To mitigate the impact of vertebral artery geometry, we have presented our findings in a stratified manner. Our results indicate that hypertension, diabetes, and the FTP are independent risk factors for the formation of BA plaques. Hypertension and diabetes contribute to atherogenesis, the progression of atherosclerotic lesions, and the overall outcomes of atherosclerosis by promoting lipid accumulation and triggering inflammatory responses (2). Furthermore, our study revealed that the incidence rate of BA plaque formation in hypertensive patients was 3.8 times higher than that observed in normotensive patients, a finding that aligns with previous research (22). It has been suggested that hypertensive patients would benefit from regular MRI examinations to assess the presence and potential risk of rupture associated with basilar artery plaques. This study is the first to identify the FTP as a morphological risk factor for BA plaque formation, with an OR of 3.196. An instance of a patient with FTP is depicted in Figure 2, showcasing a plaque at the distal end of the BA as observed on sagittal and axial HR-MRI. Previous studies have established that FTP augments the risk of posterior circulation ischemic stroke due to the impaired capacity to reestablish blood supply as a result of abnormal cerebral blood distribution (12, 23). Our findings introduce a novel perspective on the etiology of posterior ischemic stroke in FTP patients, highlighting the high prevalence of BA plaques. This insight is clinically valuable for investigating the underlying causes in patients presenting with posterior ischemic stroke.

Plaque burden is recognized as a critical factor in assessing plaque vulnerability (24, 25), directly reflecting the severity of atherosclerotic disease and holding significant value for the prevention and treatment of cerebrovascular events. The NWI is a widely accepted metric for quantifying plaque burden, considering both the VA and the WT (18). Previous studies have established that plaque burden is associated with an increased risk of ischemic stroke events, independent of the degree of stenosis alone (26, 27), so we did not include the stenosis of BA as an outcome. In the patients with Lambda geometry, the PCA morphology type was positively correlated with mean NWI and it was negatively correlated with mean LA and mean VA. These results could explain why patients with FTP tend to have a higher risk of posterior ischemic stroke. Furthermore, it was surprising that the PCA morphology type was positively correlated with the curvature of BA. Deng, et al. (28) pointed out the BA curvature was a predictive factor of BA plaque burden. Therefore, the mechanism of the correlation between the PCA morphology type and plaque burden may be the result of the BA curvature, and it may be approved by hemodynamic analysis of BA system in the next future.

Another valuable finding was that patients with CFTP and PFTP exhibited a higher incidence of posterior circulation infarction compared to those with a normal PCA, aligning with findings from a previous study (21). However, there was no significant differences between CFTP and PFTP groups. It was traditionally thought that the occurrence of posterior circulation infarction in patients with FTP was the cause of abnormal hemodynamics and perfusion in cerebral artery (29, 30). Yet, the precise relationship between these factors remains uncertain (31). Our study elucidates a potential mechanism underlying posterior circulation infarction in patients with FTP, suggesting that it may be mediated by the formation and burden of BA plaques.

Although our study has revealed significant findings, it is not without limitations. Firstly, the study was conducted at a single center, which may affect the generalizability of our results. Secondly, the sample size was relatively small, particularly for the subgroups with Walking and Tuning Fork geometries. This limited our ability to thoroughly analyze the relationship between PCA morphology and BA plaque formation. Lastly, the precise mechanisms underlying the corresponding hemodynamic changes in the vertebrobasilar system’s morphology remain unclear. Future research should focus on elucidating the hemodynamics of the vertebrobasilar artery system to address this knowledge gap.

## Conclusion

FTP was a risk factor of BA plaque formation and it was correlated with BA burden, and which could be used to explain the posterior circulation infarction in patients with FTP.

## Significance statement

FTP was an independent risk factor for BA atherosclerosis, and it was firstly mentioned in the study. The morphology of PCA was correlated with BA plaque burden, and it showed a significant evaluation on the posterior circulation ischemic stroke. Our results provided a new idea to elucidate the cause of posterior ischemic stroke in patients with FTP through the high incidence of BA plaque, and it was clinically helpful for seeking the etiology of patients with posterior ischemic stroke.

## Data Availability

The original contributions presented in the study are included in the article/supplementary material, further inquiries can be directed to the corresponding authors.
